# T-type calcium channel Ca_v_3.2 deficient mice show elevated anxiety, impaired memory and reduced sensitivity to psychostimulants

**DOI:** 10.3389/fnbeh.2014.00092

**Published:** 2014-03-18

**Authors:** Giuseppe Gangarossa, Sophie Laffray, Emmanuel Bourinet, Emmanuel Valjent

**Affiliations:** ^1^Institut de Génomique Fonctionnelle, CNRSUMR-5203, Montpellier, France; ^2^INSERMU661, Montpellier, France; ^3^Universités de Montpellier 1 and 2UMR-5203, Montpellier, France; ^4^Laboratories of Excellence, Ion Channel Science and Therapeutics, Institut de Génomique FonctionnelleMontpellier, France

**Keywords:** T-type Ca^**2+**^ channels, anxiety, memory and learning, psychostimulants, drugs of abuse, spontaneous behavior

## Abstract

The fine-tuning of neuronal excitability relies on a tight control of Ca^2+^ homeostasis. The low voltage-activated (LVA) T-type calcium channels (Ca_v_3.1, Ca_v_3.2 and Ca_v_3.3 isoforms) play a critical role in regulating these processes. Despite their wide expression throughout the central nervous system, the implication of T-type Ca_v_3.2 isoform in brain functions is still poorly characterized. Here, we investigate the effect of genetic ablation of this isoform in affective disorders, including anxiety, cognitive functions as well as sensitivity to drugs of abuse. Using a wide range of behavioral assays we show that genetic ablation of the *cacna1h* gene results in an anxiety-like phenotype, whereas novelty-induced locomotor activity is unaffected. Deletion of the T-type channel Ca_v_3.2 also triggers impairment of hippocampus-dependent recognition memories. Acute and sensitized hyperlocomotion induced by d-amphetamine and cocaine are dramatically reduced in T-type Ca_v_3.2 deficient mice. In addition, the administration of the T-type blocker TTA-A2 prevented the expression of locomotor sensitization observed in wildtype mice. In conclusion, our data reveal that physiological activity of this specific Ca^2+^ channel is required for affective and cognitive behaviors. Moreover, our work highlights the interest of T-type channel blockers as therapeutic strategies to reverse drug-associated alterations.

## Introduction

Affective and cognitive functions constitute a complex ensemble of behavioral skills strongly dependent on the fine regulation of intracellular homeostasis and neuronal excitability. The fine regulation of calcium (Ca^2+^) signaling is critical for basic neuronal functions including action potential generation, neurotransmitter release and synaptic plasticity (Berridge, 1998). Ca^2+^ is the most common signal transduction element in neurons and its entry is tightly regulated by two major classes of voltage-gated calcium channels (VGCCs): the high-voltage activated (HVA) (L-, P/Q and N-type) and the low-voltage activated (LVA) (T-type) calcium channels.

Three subtypes of T-type channel have been cloned, namely, Ca_v_3.1 (a1G), Ca_v_3.2 (a1H) and Ca_v_3.3 (a1I) (Cribbs et al., 1998, 2000; Lee et al., 1999; Zhuang et al., 2000; Gomora et al., 2002). T-type VGCCs are widely expressed throughout the body and, given their ability to switch neuronal firing patterns and modulate burst-firing activity in the central nervous system they have been implicated in several physiological aspects including sleep regulation, body weight maintenance and pain (Huguenard, 1996; Cribbs et al., 1998, 2000; Talley et al., 1999; Nilius et al., 2006; Bourinet et al., 2014). Disruption of calcium-dependent low-threshold currents mediated by T-type calcium channels have been associated with a wide range of neurological and neuropsychiatric disorders including epilepsy, insomnia, Parkinson’s disease, depression and schizophrenia as well as chronic pain syndromes (Huguenard and Prince, 1994; Kim et al., 2001, 2003; Anderson et al., 2005; Bourinet et al., 2005; Choi et al., 2007; Llinas et al., 2007; Uebele et al., 2009; Miwa et al., 2011; Francois et al., 2013; Park et al., 2013). Interestingly, several classes of drugs currently used in therapy (antipsychotics, antidepressants and antiepileptics) also show activity on T-type channels (Heady et al., 2001; Santi et al., 2002; Traboulsie et al., 2006; Kraus et al., 2007), thus suggesting T-type channels as a potential target for some brain dysfunctions. In addition, other calcium channel blockers including mibefradil, nicardipine, 2-octanol and amilodipine, which non-specifically act also on T-type channels, have been shown to reduce the psychomotor effects of cocaine, methamphetamine and phencyclidine (PCP; Popoli et al., 1993; Hori et al., 1998; Bisagno et al., 2010). More recently, the novel and specific blocker of T-type channels TTA-A2 has been reported to efficiently alleviate psychomotor effects induced by MK-801 and amphetamine as well as conditioned avoidance responding (Kraus et al., 2010; Uslaner et al., 2012), thus strengthening the strategic interest of studying the T-type channels physiology and pharmacology.

Although the implication of T-type channels in neuronal excitability has been extensively studied (Nelson et al., 2006; Shin et al., 2008; Iftinca and Zamponi, 2009; Cheong and Shin, 2013), very little is known about the role of T-type channels in motor, affective and cognitive functions. Based on the fact that Ca_v_3.2 channels are widely expressed in the basal ganglia circuit, in limbic as well as in cortical areas (Talley et al., 1999) and that such circuits are critical components for the correct computation of many of these behaviors, we therefore decided to investigate the involvement of T-type Ca_v_3.2 channels in spontaneous and drugs-altered performances. Understanding the physiological role of T-type calcium channels has been for long time limited by the lack of selective pharmacological compounds (Perez-Reyes, 2003), but new T-type antagonists have recently been reported (Barrow et al., 2007; Shipe et al., 2008; Yang et al., 2008). In this study, we deciphered the implication of Ca_v_3.2 calcium channel in anxiety and memory disorders as well as in sensitivity to psychostimulants using Ca_v_3.2 deficient mice.

## Materials and methods

### Animals

Ca_v_3.2 knockout mice (Chen et al., 2003) were maintained in the C57BL/6J background. Both WT and KO littermates were generated from Ca_v_3.2 heterozygous breeders. 8–12 weeks old male mice were used and maintained in a 12 h light/dark cycle, in stable conditions of temperature (22°C) and humidity (60%), with food and water *ad libitum*. All experiments were in accordance with the guidelines of the French Agriculture and Forestry Ministry for handling animals (D34-172-13).

### Drugs

Cocaine hydrochloride (15 mg/kg, Sigma-Aldrich) and d-amphetamine sulfate (2 mg/kg, Sigma-Aldrich) were dissolved in 0.9% physiological saline and injected intraperitoneally (i.p.) in a body volume of 10 ml/kg. The T-type blocker TTA-A2 (1 mg/kg, p.o., Merck) was dissolved in a solution containing 0.05% methylcellulose in H_2_O and 10% Tween80.

### Light/dark conflict test

The light/dark conflict test, which is believed to measure anxiety-related behavior (Crawley and Goodwin, 1980), was performed by placing the mouse in a cage (35 cm width × 50 cm length × 20 cm height) that has two chambers, one bigger and bright (2/3), and the other smaller and dark (1/3). The animal was initially placed in the lighted side, and transitions between sides and the time spent in each division were recorded for 10 min. The apparatus was wiped with 70% ethanol between sessions.

### Open field

Spontaneous exploratory behavior was monitored in an open field (white plastic arena with 35 cm width × 50 cm length × 20 cm height) for 10 min. The open field was wiped with 70% ethanol between sessions. The center zone was defined as a virtual perimeter within 5 cm from the sides of the arena. Experiments were videotaped and an observer scored the time spent in the center (four paws inside the center zone) and the number of transitions in the center zone.

### Elevated-plus maze (EPM)

The elevated-plus maze (EPM) was elevated 1 m above the floor and was constructed of black plastic with 2 open arms (5 cm width × 35 cm length × 0.5 cm height of the walls) and two closed arms (5 width × 35 cm length × 15 cm height of the walls). Mice were placed in the center of an EPM facing one of the open arms and were allowed to explore the maze for 10 min. The EPM was wiped with 70% ethanol between sessions. Experiments were videotaped and scored for entries and time spent in the closed arms (four paws within closed arm) or open arms (four paws within open arms).

### Rotarod test

Balance and motor coordination as well as motor learning were assessed using a mouse accelerating rotarod (Ugo Basile, Comerio, Italy). Mice were placed on the rotating drum that accelerated from 4 to 40 rpm over 5 min for four trials a day, for four consecutive days. The trial interval was 45 min for all the mice. Rotarod scores were scored for latency to either fall or ride the rod around.

### Grip-strength test

The grip-strength apparatus (BioSeb, Chaville, France) comprised of a wire grid (8 × 8 cm) connected to an isometric force transducer (dynamometer). Mice were lifted by the tail so that their forepaws could grasp the grid. The mice were then gently pulled backward by the tail until the grid was released. The mean of three consecutive measurements for each mouse was calculated. The muscular strength was expressed in Newton (N) as mean ± SEM.

### Horizontal grid test

A cage grid is held at least 35 cm above a mouse cage containing 5–7 cm of soft bedding. Each hang period begins with all four paws of the mouse grasping the greed. The hang time is measured from the time the grid is inverted to the time that the mouse falls off the wire grid. A fixed limit of 60 s is applied for the hanging period.

### Spontaneous and drugs-induced locomotor activity

Locomotor activity was measured as described previously (Brami-Cherrier et al., 2005; Gangarossa et al., 2013). Horizontal and vertical activity was measured in a circular corridor (Imetronic, Pessac, France). Counts for horizontal activity were incremented by consecutive interruption of two adjacent beams placed at a height of 1 cm per 90° sector of the corridor (mice moving through one-quarter of the circular corridor) and counts for vertical activity (rearings) as interruption of beams placed at a height of 7.5 cm along the corridor (mice stretching upwards). All mice were habituated to the test apparatus, handling, and procedure for three consecutive days before the actual experiment. In this habituation procedure mice were placed for 30 min in the activity box, received a first injection of saline, and were placed back in the box for 2 h. For the acute drug injection, the handling was identical, except that the saline injection was replaced by administration of cocaine (15 mg/kg, i.p.) and d-amphetamine (2 mg/kg, i.p). In cocaine and amphetamine sensitization experiments, mice were treated daily with cocaine (15 mg/kg, i.p.) and amphetamine (2 mg/kg, i.p.) for five consecutive days. This repeated exposure was followed by 8 days of withdrawal and by a challenge injection of cocaine (15 mg/kg) or d-amphetamine (2 mg/kg). Locomotor activity was measured as described above. The day after the challenge, wildtype and Ca_v_3.2 deficient mice were treated with TTA-A2 (1 mg/kg) or its vehicle 30 min prior the administration of d-amphetamine or cocaine. The degree of psychostimulants-induced locomotor sensitization was determined from the slope of the curve corresponding to the 5 days of d-amphetamine or cocaine administration.

### Spontaneous alternation Y-Maze

The Y-maze apparatus, made of Plexiglas had three identical arms (40 × 9 × 16 cm) placed at 120° with respect to each other. Each mouse was placed at the end of one arm and allowed to explore freely the apparatus for 5 min. Spontaneous alternation performance (SAP) was assessed by scoring the pattern of entries into each arm during the 5 min of the test. Alternations were defined as successive entries into each of the three arms as on overlapping triplet sets (i.e., ABC, BCA, ‥.). Percent of spontaneous alternations was defined as the ratio of actual (= total alternations) to possible (= total arm entries −2) number of alternations × 100. Total entries were scored as an index of ambulatory activity in the Y-maze.

### Novel object recognition (NOR)

Mice were habituated to a V-maze with two identical arms (34 × 6 × 15 cm, at 90°) for 10 min. The following day, mice were allowed to freely explore two objects (A and B) located at the end of the two arms. Object interaction was defined as approaching the object with the nose closer than 1 cm. Following a retention interval of 24 h, mice underwent a 5 min recall session during which the V-maze contained a familiar object (A) and a novel object (C). Following each session, the objects and the open field were cleaned with 70% ethanol. The experiments were videotaped and the time spent exploring the objects was scored. The percentage of exploration was calculated using the formula % exploration = ((Time C)/(Time A + Time C)) × 100.

### Spatial object recognition (SOR)

Mice were habituated to an arena for 10 min. The following day, mice were allowed to freely explore two different objects (A and B) located in the same site of the arena. Object interaction was defined as approaching the object with the nose closer than 1 cm. Following a retention interval of 24 h, mice underwent a 5 min recall session during which the arena contained one of the two objects displaced in the other site of the arena (novel place). Following each session, the objects and the open field were cleaned with 70% ethanol. The experiments were videotaped and the time spent exploring the objects was scored. The percentage of exploration was calculated using the formula % exploration = ((Time B)/(Time A + Time B)) × 100.

### Marble burying

Mice were placed in the center of a clear plastic cage containing 5 cm of sawdust bedding with 24 glass marbles (1.5 cm diameter) on top, arranged in four rows of six marbles. The lid of the cage was used to prevent mice from escaping. Mice were then allowed to explore the testing chamber for 20 min. The number of marbles buried at least two-thirds deep were counted.

### Statistics

The data were analyzed using one-way or two-way ANOVA followed by Bonferroni *post-hoc* test for specific comparisons. Student’s *t*-test with equal variances was used for groups of two, when relevant. In all cases, significance threshold was set at *p* < 0.05. Statistical analyses were performed using GraphPad Prism 5.0 (GraphPad Prism Software Inc., San Diego, USA).

## Results

### Novelty-induced spontaneous locomotor activity is not impaired in Ca_v_3.2 deficient mice

**Figure 1:**
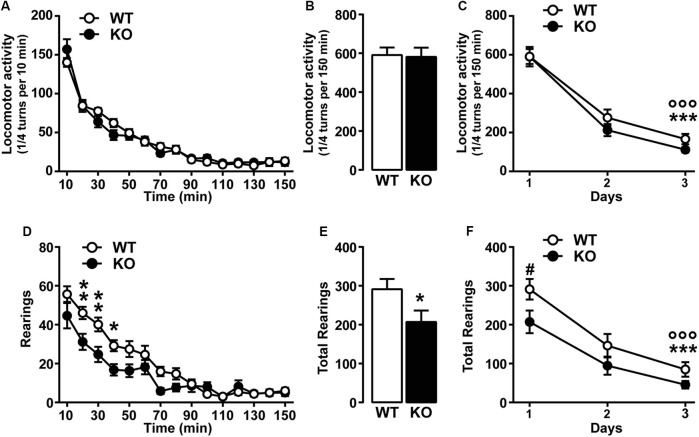
**Spontaneous locomotor activity in Ca_v_3.2 deficient mice. (A)** Horizontal locomotor activity of Ca_v_3.2 null mice (*n* = 22) and their WT littermates (*n* = 26) in a novel non-stressful environment. Data (means ± SEM) were analyzed using two-way ANOVA: (Time × Genotype: *F*_(14, 644)_ = 1.607, *P* = 0.0724; Time: *F*_(14, 644)_ = 157.8, *P* < 0.0001; Genotype: *F*_(1, 46)_ = 0.0268, *P* = 0.8707). **(B)** Cumulative locomotor activity of WT and Ca_v_3.2 KO mice over a 150 min period. Data (means ± SEM) were analyzed using Student’s *t*-test: NS. **(C)** Environmental habituation over a period of 3 days measured as a reduction of spontaneous horizontal locomotor activity. Data (means ± SEM) were analyzed using two-way ANOVA: (Time × Genotype: *F*_(2, 92)_ = 0.8412, *P* = 0.4345; Time: *F*_(2, 92)_ = 161.4, *P* < 0.0001; Genotype: *F*_(1, 46)_ = 0.9561, *P* = 0.3333). Specific comparisons: *** *p* < 0.001 (WT-Day3 *vs.* WT-Day1) and °°° *p* < 0.001 (KO-Day3 *vs.* KO-Day1). **(D)** Rearing activity (vertical locomotor activity) of Ca_v_3.2 null mice (*n* = 22) and their WT littermates (*n* = 26) in a novel non-stressful environment. Data (means ± SEM) were analyzed using two-way ANOVA: (Time × Genotype: *F*_(14, 644)_ = 3.751, *P* < 0.0001; Time: *F*_(14, 644)_ = 70.86, *P* < 0.0001; Genotype: *F*_(1, 46)_ = 4.601, *P* = 0.0373). Specific comparisons: ** *p* < 0.01 and * *p* < 0.05 (KO *vs.* WT). **(E)** Cumulative rearing activity of WT and Ca_v_3.2 KO mice over a 150 min period. Data (means ± SEM) were analyzed using Student’s *t*-test: * *p* < 0.05. **(F)** Environmental habituation over a period of 3 days measured as a reduction of spontaneous rearing activity. Data (means ± SEM) were analyzed using two-way ANOVA: (Time × Genotype: *F*_(2, 92)_ = 1.139, *P* = 0.3246; Time: *F*_(2, 92)_ = 76.78, *P* < 0.0001; Genotype: *F*_(1, 46)_ = 3.874, *P* = 0.05). Specific comparisons: *** *p* < 0.001 (WT-Day3 *vs.* WT-Day1), °°° *p* < 0.001 (KO-Day3 *vs.* KO-Day1) and ^#^ *p* < 0.05 (KO-Day1 *vs.* WT-Day1).

We first investigated the spontaneous locomotor activity of Ca_v_3.2 deficient mice in response to novelty, in a non-stressful environment (low luminosity). No differences in the initial horizontal activity or in the habituation phase (3 days) were observed between WT and Ca_v_3.2 KO mice (Figures 1A–C), thus suggesting intact exploratory drive. In addition, despite a mild but significant attenuation in vertical locomotor activity (rearing behavior) observed in Ca_v_3.2 deficient mice during the first day (Figures 1D and E), both genotypes showed habituation to the testing environment (Figure 1F).

To investigate whether the genetic ablation of *Cacna1h* gene might induce muscular alteration that can ultimately impact on the spontaneous behavior of mice, we assessed muscular strength using two essays: the grip-strength and the grid test. Both tests revealed no major differences between genotypes. Grip-strength test: WT (1.68 N ± 0.076, *n* = 15) and KO (1.85 N ± 0.084, *n* = 14). Grid test: WT (59.87 sec ± 0.13, *n* = 15) and KO (59.79 sec ± 0.15, *n* = 14).

Taken together our results show that *Cacna1h* gene deletion does not alter spontaneous locomotor performance and exploratory drive.

### Ca_v_3.2 deficient mice show elevated anxiety

**Figure 2:**
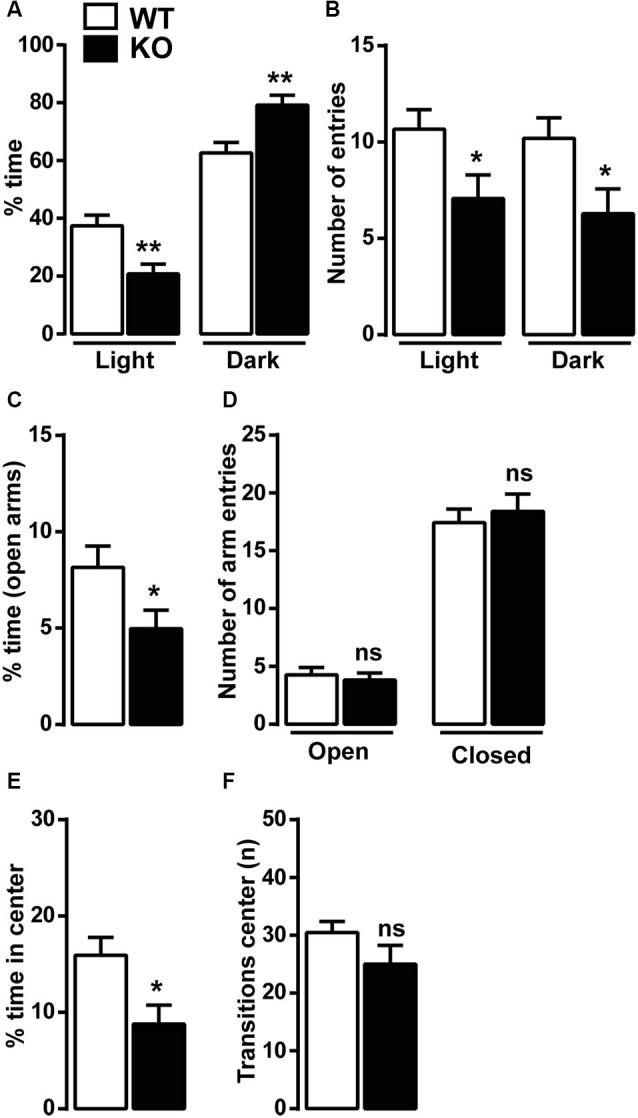
**Anxiety-like behavior in Ca_v_3.2 deficient mice.** Mice were tested in three anxiety-related behavioral paradigms: the ligh/dark conflict test, the elevated plus maze test (EPM) and the open field test. **(A)** Histograms show the percentage of time Ca_v_3.2 KO (*n* = 14) and WT mice (*n* = 15) spent in the light and the dark compartments. **(B)** Histograms indicate the number of entries of Ca_v_3.2 KO (*n* = 14) and WT mice (*n* = 15) in the light and the dark compartments. **(A** and **B)** Data (means ± SEM) were analyzed using Student’s *t*-test: * *p* < 0.05; ** p < 0.01. **(C)** Histograms show the percentage of time Ca_v_3.2 KO (*n* = 17) and WT mice (*n* = 19) spent in the open arms of the EPM. Data (means ± SEM) were analyzed using Student’s *t*-test: * *p* < 0.05. **(D)** Histograms indicate the number of entries of Ca_v_3.2 KO (*n* = 17) and WT mice (*n* = 19) in open and closed arms of EPM. Data (means ± SEM) were analyzed using student’s *t*-test: ns. **(E)** Histograms show the percentage of time Ca_v_3.2 KO (*n* = 14) and WT mice (*n* = 15) spent in the center zone of the open field. Data (means ± SEM) were analyzed using student’s *t*-test: * *p* < 0.05. **(F)** Histograms indicate the number of transitions Ca_v_3.2 KO (*n* = 14) and WT mice (*n* = 15) made in the center zone of the open field. Data (means ± SEM) were analyzed using student’s *t*-test: ns.

Because of the reduced vertical locomotor activity, we next investigated anxiety-related behaviors in Ca_v_3.2 deficient mice using well-established behavioral paradigms: the light/dark conflict context, the EPM and the open field tests. In the light/dark test, KO mice spent less time in the light compartment and more time in the dark compartment compared to WT mice (Figure 2A). Ca_v_3.2 deficient mice were also slightly less active than WT as demonstrated by the reduced number of entries in both compartments (light and dark) (Figure 2B). In the EPM test, Ca_v_3.2 deficient mice spent less time in the open arms compared to their WT littermates confirming an increased anxiety-related phenotype (Figure 2C). No differences were observed in the number of entries in both closed and open arms suggesting that this effect is not the result of an altered locomotor response (Figure 2D). To further extend these behavioral observations, we measured the spontaneous behavior of Ca_v_3.2 deficient mice to explore a novel and stressful environment using the open field test. As shown in Figure 2E, Ca_v_3.2 deficient mice spent less time in the center of the arena compared to their WT littermates and made an equal number of transitions in the center zone compared to WT mice therefore indicating increased anxiety rather than a general locomotor deficit (Figure 2F).

Anxiety disorders are often exacerbated by repetitive and compulsive behaviors, such as in obsessive-compulsive disorder (OCD), in which abnormal repetitive behaviors can take place to alleviate recurrent and generalized anxiety. The marble burying test was then performed to assess whether Ca_v_3.2 null mice displayed some forms of compulsivity. No differences were observed in the number of marbles buried: WT (15.15 ± 1.5, *n* = 13) and KO (16.31 ± 0.5, *n* = 13).

Altogether these data strongly suggest that T-type Ca_v_3.2 channel influence anxiety-related behaviors, which do not seem being associated with repetitive and compulsive behaviors. Further studies should be performed to investigate whether T-type channels participate to altered behaviors observed in OCD.

### Recognition memory functions are disrupted Ca_v_3.2 null mice

**Figure 3:**
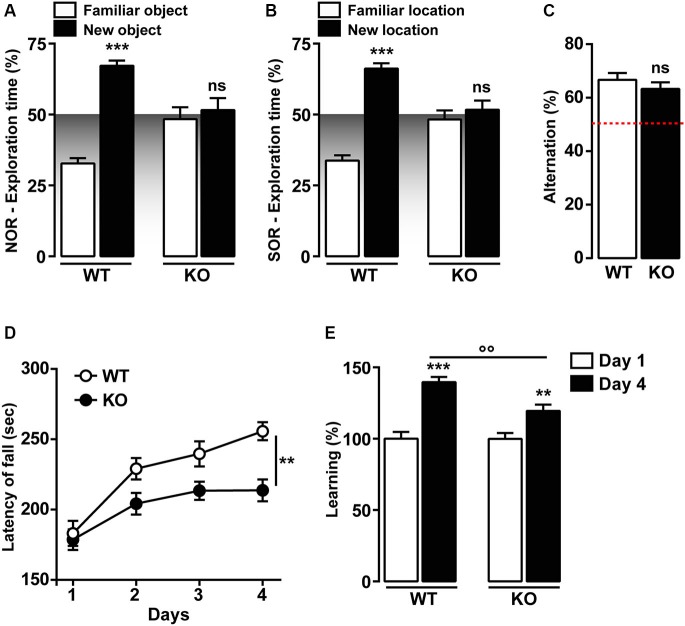
**Memory functions in Ca_v_3.2 deficient mice. (A)** Graph shows the percentage of exploration time of Ca_v_3.2 KO (*n* = 14) and WT (*n* = 15) mice in the NOR test. Data (means ± SEM) were analyzed using two-way ANOVA: (Object exploration × Genotype: *F*_(1, 44)_ = 25.80, *P* < 0.0001; Object exploration: *F*_(1, 44)_ = 37.58, *P* < 0.0001; Genotype: *F*_(1, 44)_ = 3.245e-014,* P* = 0.99). Specific comparisons: *** *p* < 0.001 (WT-new *vs.* WT-familiar). **(B)** Graph shows the percentage of exploration time of Ca_v_3.2 KO (*n* = 14) and WT (*n* = 15) mice in the SOR test. Data (means ± SEM) were analyzed using two-way ANOVA: (Object exploration × Genotype:* F*_(1, 44)_ = 31.80, *P* < 0.0001; Object exploration: *F*_(1, 44)_ = 48.81, *P* < 0.0001; Genotype: *F*_(1, 44)_ = 1.324e-013, *P* = 0.99). Specific comparisons: *** *p* < 0.001 (WT-new *vs.* WT-familiar). **(C)** Histogram indicates the percentage of spontaneous alternation of Ca_v_3.2 KO (*n* = 14) and WT (*n* = 15) in the Y-maze test. The red dotted line represents the chance level of alternation (random, 50%). Data (means ± SEM) were analyzed using Student’s *t*-test: ns. **(D)** Curves show coordination and motor learning in WT (*n* = 22) and Ca_v_3.2 KO (*n* = 22) mice over a training period of 4 days. Data (means ± SEM) were analyzed using two-way ANOVA (Time × Genotype: *F*_(3, 168)_ = 2.017, *P* = 0.1134; Time: *F*_(3, 168)_ = 19.03, *P* < 0.0001; Genotype: *F*_(1, 168)_ = 20.12, *P* < 0.0001). Specific comparisons: ** *p* < 0.01 (KO-Day4 *vs.* WT-Day4). **(E)** Percentage of motor learning in WT (*n* = 22) and Ca_v_3.2 KO (*n* = 22). Data (means ± SEM) were analyzed using two-way ANOVA (Time × Genotype: *F*_(1, 84)_ = 5.643, *P* < 0.05; Time: *F*_(1, 84)_ = 5.654, *P* < 0.05; Genotype: *F*_(1, 84)_ = 49.11, *P* < 0.0001). Specific comparisons: *** *p* < 0.001 (WT-Day4 *vs.* WT-Day1), ** *p* < 0.01 (KO-Day4 *vs.* KO-Day1) and °° *p* < 0.01 (KO-Day4 *vs.* WT-Day4).

Previous studies have shown that T-type Ca_v_3.2 is critical for hippocampal LTP, cued-context fear conditioning and passive avoidance tasks (Chen et al., 2012). We then investigated two hippocampal recognition memory tasks, the novel and the spatial object recognition (NOR and SOR, respectively). As shown in Figures 3A, B, Ca_v_3.2 deficient mice do not show preference for the novel or the relocated object during the recall session 24 h later as opposed to the WT. This altered response is not the result of an impairment of the exploratory drive since the exploration of the two objects is not significantly different from the WT animal during the familiarization phase (data not shown). Interestingly, the spatial working memory, assessed using the spontaneous alternation in the Y-Maze, was unaffected in Ca_v_3.2 deficient mice (Figure 3C).

Motor skill learning was also investigated in Ca_v_3.2 deficient mice using the accelerating rotarod. No differences were observed during the first day of rotarod training, thus suggesting unaltered motor coordination in Ca_v_3.2 deficient mice (Figures 3D, E). Although Ca_v_3.2 KO mice showed a significant reduction of the motor performance compared to their WT littermates (Figures 3D, E), both genotypes gradually acquired motor skills following extensive training (Figures 3D, E).

Altogether, these data strongly suggest that T-type Ca_v_3.2 channels influence preferentially memory recognition tasks, but spares working memory and motor learning.

### Psychomotor effects of d-amphetamine and cocaine are reduced in Ca_v_3.2 deficient mice

**Figure 4:**
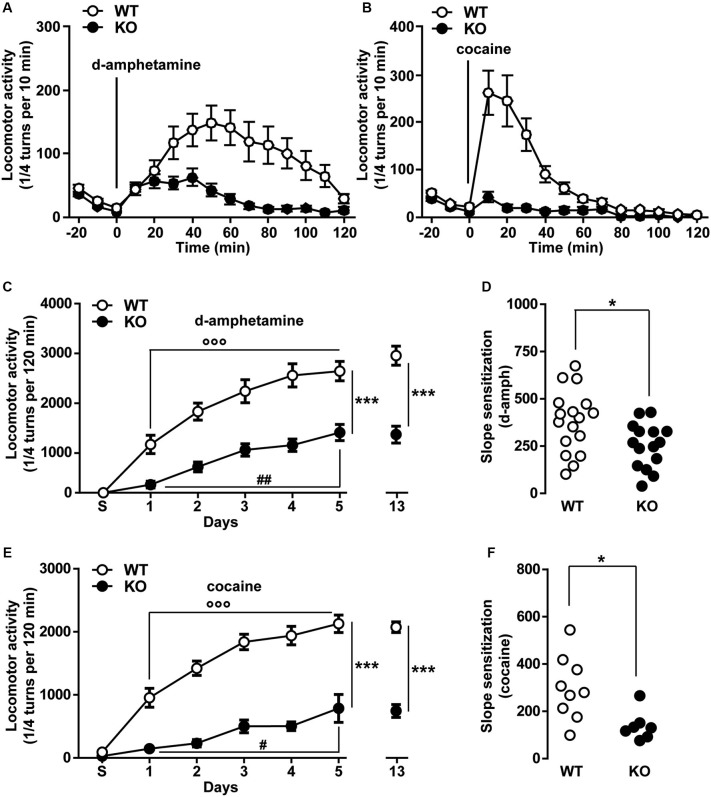
**Psychomotor effects of d-amphetamine and cocaine in Ca_v_3.2 deficient mice. (A)** Locomotor activity induced by single administration of d-amphetamine (2 mg/kg, i.p.) in WT (*n* = 17) and Ca_v_3.2 KO (*n* = 15) mice. Data (means ± SEM) were analyzed using two-way ANOVA: (Treatment × Genotype: *F*_(14, 420)_ = 4.805, *P* < 0.0001; Treatment: *F*_(14, 420)_ = 8.34, *P* < 0.0001; Genotype: *F*_(1, 30)_ = 15.85, *P* < 0.001). **(B)** Locomotor activity induced by single administration of cocaine (15 mg/kg, i.p.) in WT (*n* = 9) and Ca_v_3.2 KO (*n* = 7) mice. Data (means ± SEM) were analyzed using two-way ANOVA: (Treatment × Genotype: *F*_(14, 196)_ = 12.09, *P* < 0.0001; Treatment: *F*_(14, 196)_ = 17.53, *P* < 0.0001; Genotype: *F*_(1, 14)_ = 20.92, *P* < 0.001). **(C)** Locomotor activity induced by repeated administration (sensitization protocol) of d-amphetamine (2 mg/kg, i.p.) in WT (*n* = 17) and Ca_v_3.2 KO (*n* = 15). Data (means ± SEM) were analyzed using two-way ANOVA: (Treatment × Genotype: *F*_(6, 210)_ = 5.280, *P* < 0.001; Treatment: *F*_(6, 210)_ = 43.34, *P* < 0.0001; Genotype: *F*_(1, 210)_ = 147.3, *P* < 0.001). Specific comparisons: *** *p* < 0.001 (KO *vs.* WT), °°° *p* < 0.001 (WT-Day5 *vs.* WT-Day1) and ^##^ *p* < 0.01 (KO-Day5 *vs.* KO-Day1). **(D)** Degree of d-amphetamine-induced sensitization measured as a slope of sensitization curves in WT (*n* = 17) and Ca_v_3.2 KO (*n* = 15). Data (means ± SEM) were analyzed using Student’s *t*-test: * *p* < 0.05. **(E)** Locomotor activity induced by repeated administration (sensitization protocol) of cocaine (15 mg/kg, i.p.) in WT (*n* = 9) and Ca_v_3.2 KO (*n* = 7). Data (means ± SEM) were analyzed using two-way ANOVA: (Treatment × Genotype: *F*_(6, 98)_ = 9.086, *P* < 0.0001; Treatment: *F*_(6, 98)_ = 37.96, *P* < 0.0001; Genotype: *F*_(1, 98)_ = 301.3, *P* < 0.0001). Specific comparisons: *** *p* < 0.001 (KO *vs.* WT), °°° *p* < 0.001 (WT-Day5 *vs.* WT-Day1) and ^#^ *p* < 0.05 (KO-Day5 *vs.* KO-Day1). **(F)** Degree of cocaine-induced sensitization measured as a slope of sensitization curves in WT (*n* = 9) and Ca_v_3.2 KO (*n* = 7). Data (means ± SEM) were analyzed using student’s *t*-test: * *p* < 0.05.

The psychomotor effects of cocaine, methamphetamine and PCP have been shown to partly involve the activity of T-type calcium channels (Popoli et al., 1993; Hori et al., 1998; Bisagno et al., 2010). To test the specific contribution of T-type Ca_v_3.2 channels in these locomotor responses, the acute hyperlocomotion induced by d-amphetamine (2 mg/kg, i.p.) and cocaine (15 mg/kg, i.p.) was evaluated in WT and Ca_v_3.2 deficient mice. As shown in Figure 4A, the total locomotor activity over a 120 min period induced by a single injection of d-amphetamine was dramatically reduced in Ca_v_3.2 deficient mice compared to their WT littermates. A similar effect was also observed when mice were treated with cocaine (Figure 4B).

We next determine whether Ca_v_3.2 KO mice could still undergo locomotor sensitization despite the reduced acute locomotor response. Repeated exposure to d-amphetamine (2 mg/kg) results in a progressive and persistent enhancement of the locomotor response in WT mice (Figure 4C). Ca_v_3.2 deficient mice also displayed locomotor sensitization but this response remained lower than in WT mice even when the mice were challenged after a withdrawal period of 8 days (Figure 4C). Interestingly, taking into account the slope sensitization, the locomotor sensitization to d-amphetamine was less pronounced in Ca_v_3.2 deficient mice than in their WT controls (Figure 4D). Similar results have been obtained when mice were repeatedly injected with cocaine (Figures 4E, F). Altogether, these results show that Ca_v_3.2 null mice displayed a reduction of sensitized responses to d-amphetamine and cocaine.

**Figure 5:**
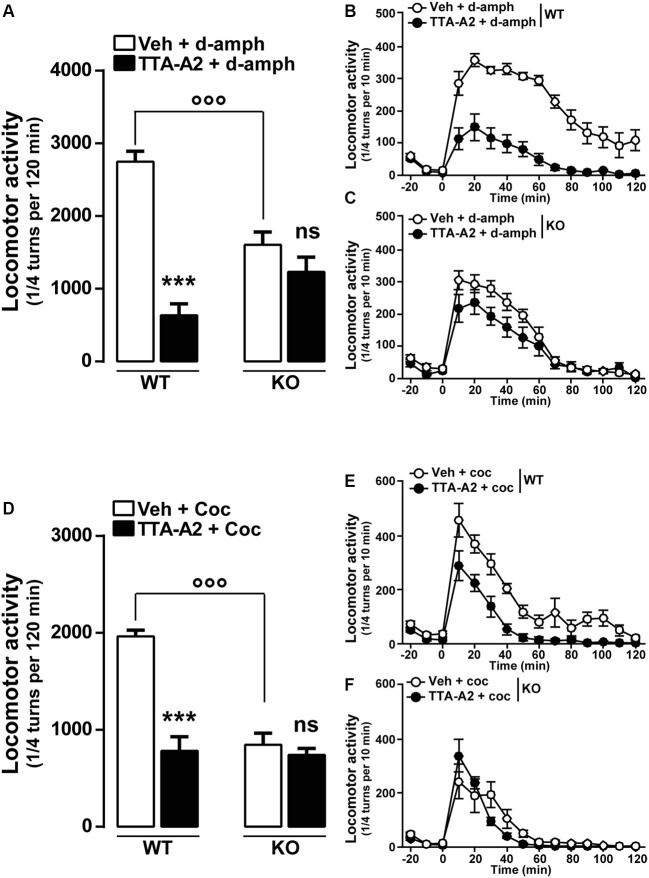
**Effect of the T-type calcium channel inhibitor TTA-A2 on psychostimulants-induced locomotor activity. (A)** Histograms show the effect of TTA-A2 (1 mg/kg, p.o.) on d-amphetamine-sensitized mice. Data (means ± SEM) were analyzed using two-way ANOVA: **A** (Treatment × Genotype: *F*_(1, 28)_ = 26.47, *P* < 0.0001; Treatment: *F*_(1, 28)_ = 2.595, *P* = 0.1185; Genotype: *F*_(1, 28)_ = 53.99, *P* < 0.0001). Specific comparisons: *** *p* < 0.001 (WT-TTA-A2+d-amph *vs.* WT-Veh+d-amph) and °°° *p* < 0.001 (KO-Veh+d-amph *vs.* WT-Veh+d-amph). **(B** and **C)** Curves showing the behavioral profile of T-type channels blocker TTA-A2 in WT (**B**, *n* = 9) and Ca_v_3.2 KO (**C**, *n* = 7) d-amphetamine-treated mice. Data (means ± SEM) were analyzed using two-way ANOVA: **B** (Treatment × Time: *F*_(14, 224)_ = 9.302, *P* < 0.0001; Time: *F*_(14, 224)_ = 34.60, *P* < 0.0001; Treatment: *F*_(1, 16)_ = 83.58, *P* < 0.0001); **C** (Treatment × Time: *F*_(14, 168)_ = 2.717, *P* < 0.01; Time: *F*_(14, 168)_ = 85.72, *P* < 0.0001; Treatment: *F*_(1, 12)_ = 2.108, *P* = 0.1722). **(D)** Histograms show the effect of TTA-A2 (1 mg/kg, p.o.) on cocaine-sensitized mice. Data (means ± SEM) were analyzed using two-way ANOVA: **A** (Treatment × Genotype: *F*_(1, 28)_ = 24.15,* P* < 0.0001; Treatment: *F*_(1, 28)_ = 27.96, *P* < 0.0001; Genotype: *F*_(1, 28)_ = 34.54, *P* < 0.0001). Specific comparisons: *** *p* < 0.001 (WT-TTA-A2+Coc *vs.* WT-Veh+Coc) and °°° *p* < 0.001 (KO-Veh+Coc *vs.* WT-Veh+Coc). **(E** and **F)** Curves showing the behavioral profile of T-type calcium channels blocker TTA-A2 in WT (**E**, *n* = 9) and Ca_v_3.2 KO (**F**, *n* = 7) cocaine-treated mice. Data (means ± SEM) were analyzed using two-way ANOVA: **E** (Treatment × Time: *F*_(14, 224)_ = 2.31, *P* < 0.01; Time: *F*_(14, 224)_ = 37.34, *P* < 0.0001; Treatment: *F*_(1, 16)_ = 50.35, *P* < 0.0001); **F** (Treatment × Time: *F*_(14, 168)_ = 1.768, *P* < 0.05; Time: *F*_(14, 168)_ = 29.27, *P* < 0.0001; Treatment: *F*_(1, 12)_ = 0.8157, *P* = 0.3842).

Finally, we examined whether a pharmacological acute blockade of T-type Ca^2+^ channel could affect the expression of locomotor sensitization in WT animals and therefore recapitulate the phenotype of Ca_v_3.2 null mice. To test this possibility, WT mice sensitized to d-amphetamine (Figures 5A–B) or cocaine (Figures 5D–E) were challenged with either vehicle or TTA-A2 (1 mg/kg, p.o.), a specific but non-selective T-type blocker prior to the last psychostimulant treatment (day 14). TTA-A2 blocked the expression of d-amphetamine and cocaine locomotor sensitization (Figures 5A, B, D, E). When the identical treatment regimen was performed in sensitized Ca_v_3.2 deficient mice, TTA-A2 had no effect on the expression of either d-amphetamine or cocaine locomotor sensitization (Figures 5A, C, D, F). Taken together, these results suggest that the effect of this T-type Ca^2+^ channel blocker on the expression of sensitization observed in WT mice was most likely attributable to the specific inhibition of the Ca_v_3.2 isoform.

## Discussion

In the present study, we evaluate the effect of genetic ablation of the *Cacna1h* gene in several behavioral paradigms related to motor, affective and cognitive functions. T-type calcium channels mediate calcium-dependent low-threshold currents and they are associated with rhythmic burst-firing activities of some brain regions (Beurrier et al., 1999; Park et al., 2010; Tai et al., 2011; Bourinet et al., 2014). By regulating Ca^2+^ homeostasis and neuronal activation, T-type calcium channels (Ca_v_3.1, Ca_v_3.2 and Ca_v_3.3) represent an interesting and promising pharmacological target. T-type calcium channels are widely expressed in the brain (Talley et al., 1999) and the T-type Ca_v_3.2 isoform is found in key regions involved in affective and cognitive behaviors. However, due to the lack of selective compounds for each T-type isoforms, little is known about their respective specific implications in the above-mentioned functions. Here, we focused on studying the T-type Ca_v_3.2 channel using Ca_v_3.2 deficient mice. Our findings reveal that the lack of *Cacna1h* gene promotes anxiety-related behavior, impairs learning and memory formation and results in a reduced sensitivity to psychostimulants (d-amphetamine and cocaine).

The involvement of T-type calcium channels in anxiety-related disorders is poorly documented. Our study reveals that mice lacking the T-type Ca_v_3.2 showed elevated anxiety across a range of behavioral tests including the light/dark conflict test, the EPM and the open field. These phenotypes were not the result of a general motor impairment since Ca_v_3.2 deficient showed unaltered locomotor response when placed in a novel environment (see also Choi et al., 2007). However, our results contrast with this previous study reporting a lack of anxiety-related phenotype in Ca_v_3.2 KO mice in the light/dark conflict test (Choi et al., 2007). Different parameters could explain this apparent discrepancy. First, the genetic background of the Ca_v_3.2 deficient mice employed in both studies was different. While our mice were on a C57BL/6J background the previous study used mice on a mixed 129/sv and C57BL/6J background (Choi et al., 2007). C57BL/6 and 129 mouse lines are the most commonly used background strains in research. However, these two strains show markedly differences in learning and memory, anxiety and sensitivity to drugs (Homanics et al., 1999; Contet et al., 2001; Cook et al., 2001, 2002; Murphy et al., 2001; Bouwknecht and Paylor, 2002). It is worth to mention that, in the light/dark conflict test, C57BL/6 show higher transition frequency and reduced time in the dark compartment compared to the 129 mouse line (Bouwknecht and Paylor, 2002). The second aspect is related to the behavioral procedure used to perform the light/dark conflict test. In the present study, mice were always placed in the light compartment at the beginning of the session (Crawley and Goodwin, 1980; Gao and Cutler, 1992; Bourin and Hascoët, 2003). This contrasts with the study of Choi and colleagues and could explain why different results have been observed between the two studies (Choi et al., 2007). Furthermore, the behavioral responses we observed in the EPM and the open field test strongly support an implication of Ca_v_3.2 T-type calcium channels in anxiety-related behaviors. Even though our results suggest that the an-xious phenotype observed in KO mice is not associated with repetitive behavior, a study clearly aiming at deciphering the role of T-type calcium channels in OCD-like behavior need to be undertaken.

Calcium-dependent signaling is important for LTP and learning and memory functions. NMDA-independent influx of Ca^2+^ through VGCCs has been shown to be crucial for both hippocampus- and amygdala-dependent tasks. Thus, HVA L-type Ca_v_1.2 and Ca_v_1.3 mutant mice showed altered spatial memory and impaired consolidation of fear conditioning, respectively (Moosmang et al., 2005; McKinney and Murphy, 2006; White et al., 2008). In addition, ablation of HVA P/Q-type Ca^2+^ channels impaired memory functions (Mallmann et al., 2013). Interestingly, disruption of the T-type calcium channel activity has been reported to strongly alter physiological induction and maintenance of LTP in the hippocampus, visual cortex and cerebellum (Yoshimura et al., 2008; Chen et al., 2012; Ly et al., 2013) and mutations of the human *cacna1h* gene have been associated with autism spectrum disorder (ASD; Splawski et al., 2006). Moreover, the demonstration that T-type calcium channels are interacting with the neurotransmitter release machinery reinforces the notion of their impact on synaptic transmission (Tang et al., 2011; Weiss et al., 2012a,b). Our study further highlights the emerging role of Ca_v_3.2 channels in learning and memory functions. Thus, while the working memory function is spared, memory recognition assessed by using the NOR and SOR is strongly impaired in Ca_v_3.2 deficient mice. This impairment is not the result of a deficit of exploratory drive since Ca_v_3.2 null mice and WT animals showed similar object exploration during the familiarization phases of the two behavioral tasks. In contrast, in the Morris water maze, memory formation and retrieval are not altered in Ca_v_3.2 deficient mice (Chen et al., 2012). In addition, we also found that, although less efficient, Ca_v_3.2 null mice were able to acquired motor skills suggesting the neural systems involved in these processes are functional. Altogether these functional data strongly suggest that T-type Ca_v_3.2 channels influence a subset of specific learning and memory processes.

The contribution of VGCCs in the action of psychostimulant drugs has been extensively documented (Karler et al., 1991; Ansah et al., 1993; Schechter, 1993; Pierce and Kalivas, 1997; Pierce et al., 1998; Licata et al., 2000; Newton et al., 2004, 2008; Rajadhyaksha and Kosofsky, 2005; Chartoff et al., 2006; Bisagno et al., 2010; Giordano et al., 2010). However, little is known about the contribution of T-type calcium channels. Here, we found that T-type Ca_v_3.2 channels are necessary for acute psychomotor effects induced by cocaine and d-amphetamine. These data extend previous results obtained in rats showing that the pharmacological blockade of T-type calcium channels achieved with TTA-A2, strongly reduced d-amphetamine-induced psychomotor effects (Bisagno et al., 2010; Uslaner et al., 2012). Interestingly, this initial study suggested that this effect would result from an alteration of the glutamatergic transmission since TTA-A2 inhibits glutamate release in the nucleus accumbens leaving unaltered dopamine influx (Uslaner et al., 2012). However, functional coupling of T-type currents to calcium-activated-potassium channels has been proposed to modify firing pattern of midbrain DA neurons (Wolfart and Roeper, 2002), a mechanism that may contribute to reward-based processes.

Our study also highlights that T-type Ca_v_3.2 channels participate to psychostimulant-induced development and expression of drug sensitization. Interestingly, previous studies pointed out that Ca_v_1.3 L-type calcium channels (LTCCs) mediate development of sensitization whereas Ca_v_1.2 LTCCs mediate expression of the psychostimulant-induced sensitized response (Giordano et al., 2010). Furthermore, our pharmacological experiment, using TTA-A2, strongly suggests that the Ca_v_3.2 channel is the only member of the T-type family required for the expression of sensitization, a crucial finding for future relevance in clinical treatments of addictions. Given the high expression of Ca_v_3.2 mRNA in the striatum, the nucleus accumbens and other nuclei that composed the rewarding systems (Talley et al., 1999), it is tempting to speculate that the effects observed are associated with altered functions of this T-type isoform within the circuit. Future experiments are necessary to identify whether the altered acute responses are associated with the function of the Ca_v_3.2 channel in a particular cell-type of the system mentioned above.

Follow up explorations would be needed in the next future to precise the cellular basis of the underlying circuits involved, especially when cell and tissue specific conditional knockout mice will be available. Moreover, elucidation of the cellular and subcellular Ca_v_3.2 expression patterns is still pending to the availability of faithful antibodies. We should also mention that brain regions (i.e., cortex, hippocampus, striatum, midbrain) known to be important for the behaviors here explored express high level of Ca_v_3.2 transcripts (Talley et al., 1999), and that functional presence of Ca_v_3.2 like T-type currents is well established in these regions (Perez-Reyes, 2003).

Our study shows that T-type Ca_v_3.2 calcium channels are strongly involved in affective and cognitive functions. In depth behavioral characterization of Ca_v_3.2 deficient mice revealed an anxiety-like phenotype although general exploratory drive was unaltered. We further demonstrate an impairment of recognition memory functions but intact working memory processing in Ca_v_3.2-ablated mice. In addition, we also provide evidence for an involvement of specific T-type Ca_v_3.2 calcium channels in sensitization processes triggered by commonly abused psychostimulant drugs.

However, it should be mentioned that mechanisms of functional compensations by other T-type calcium channels might occur due to global knockout of Ca_v_3.2 although no compensation in Ca_v_3.1 and Ca_v_3.3 has been detected at the level of the hippocampus (see GEO^1^).

In conclusion, our data highlight that physiological activity of the specific T-type Ca_v_3.2 calcium channel is required for affective and cognitive behaviors suggesting potential functional implications of this calcium channel in many brain disorders. The emergence of T-type selective pharmacology is therefore very promising and holds many clinical perspectives.

## Conflict of interest statement

The authors declare that the research was conducted in the absence of any commercial or financial relationships that could be construed as a potential conflict of interest.

## Footnotes

^1^www.ncbi.nlm.nih.gov/geo/query/acc.cgi?acc=GSE30846
